# Esketamine nasal spray shows higher remission and response rates over 32 weeks of treatment compared with quetiapine extended-release in patients with treatment resistant depression: Results from ESCAPE-TRD, a randomised, phase IIIb clinical trial

**DOI:** 10.1192/j.eurpsy.2023.272

**Published:** 2023-07-19

**Authors:** A. Reif, A. E. Anıl Yağcıoğlu, A. Luts, T. Messer, R. Nielsen, J. Buyze, T. Ito, Y. Kambarov, S. Mulhern Haughey, B. Rive, I. Usankova, C. von Holt, Y. Godinov

**Affiliations:** 1 Department of Psychiatry, Psychosomatic Medicine and Psychotherapy, University Hospital Frankfurt, Frankfurt, Germany; 2 Department of Psychiatry, Hacettepe University Faculty of Medicine, Ankara, Türkiye; 3 ProbarE in Lund AB, Lund, Sweden; 4 Danuvius Klinik GmbH, Technische Universität München, Pfaffenhofen an der Ilm, Germany; 5 Aalborg University Hospital, Aalborg, Denmark; 6 Janssen Pharmaceutica NV, Beerse, Belgium; 7 Janssen EMEA, High Wycombe, United Kingdom; 8 Janssen EMEA, Beerse, Belgium; 9 Janssen EMEA, Dublin, Ireland; 10 Janssen EMEA, Paris, France; 11 Janssen EMEA, Moscow; 12 Janssen EMEA, Neuss, Germany; 13 Janssen EMEA, Sofia, Bulgaria

## Abstract

**Introduction:**

Treatment resistant depression (TRD) is estimated to affect 10–30% of patients with major depressive disorder (Al‑Harbi *et al.* Patient Prefer Adherence 2012; 6 369–88). Esketamine nasal spray (NS), in combination with a selective serotonin reuptake inhibitor (SSRI) or serotonin norepinephrine reuptake inhibitor (SNRI), increases remission and response rates in patients with TRD compared with placebo plus SSRI/SNRI (Popova *et al*. Am J Psychiatry 2019; 176 428–38). ESCAPE-TRD (NCT04338321) is the first randomised clinical trial to compare esketamine NS to quetiapine extended-release (XR), an antipsychotic augmentation therapy for patients with TRD.

**Objectives:**

To explore the efficacy and safety of esketamine NS compared with quetiapine XR in TRD over 32 weeks (wks).

**Methods:**

In the ESCAPE-TRD phase IIIb open-label, rater-blinded trial, patients were randomised 1:1 to esketamine NS (56/84 mg; twice per wk, weekly or every 2 wks) or quetiapine XR (150–300 mg daily) both in combination with an ongoing SSRI/SNRI. Remission (Montgomery-Åsberg Depression Rating Scale [MADRS] total score of ≤10) and response (≥50% improvement in MADRS total score from baseline or MADRS≤10) rates were analysed over time using last observation carried forward. MADRS change from baseline was analysed using Mixed Models for Repeated Measures (MMRM). The most common adverse events (AEs) leading to discontinuation are reported for patients who received ≥1 dose of study medication.

**Results:**

At baseline, 336 patients were randomised to esketamine NS and 340 to quetiapine XR. A significantly higher percentage of patients in the esketamine NS group achieved remission (at each visit from Wk6 [p=0.008] onward) and response (at each visit from Day 15 [p<0.001] onward) versus patients treated with quetiapine XR. Esketamine NS significantly improved MADRS score compared to quetiapine XR at each visit from Day 8 onwards, with an average difference over time in the least squares means total MADRS score change from baseline of -2.4 (**Figure**). The most common AEs leading to treatment discontinuation for esketamine NS were dizziness (n=2, 0.6%), dissociation (n=2, 0.6%) and vomiting (n=2, 0.6%), and for quetiapine XR were sedation (n=7, 2.1%), weight increased (n=6, 1.8%) and somnolence (n=5, 1.5%).

**Image:**

**Figure:**
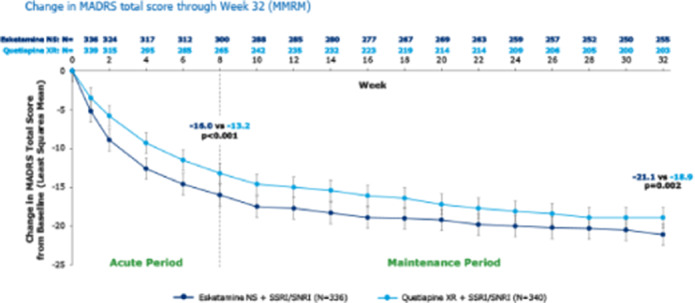

**Conclusions:**

Esketamine NS increased the percentage of patients achieving response and remission and improved MADRS total score over time compared with quetiapine XR. Rates of discontinuation arising from the most common AEs were generally lower with esketamine NS than quetiapine XR.

**Acknowledgements:**

We thank participating patients and all who assisted with the study. This study was funded by Janssen; medical writing support was provided by Carolyn Walsh, PhD, Costello Medical, UK.

**Disclosure of Interest:**

A. Reif Grant / Research support from: Medice, Consultant of: National Care Guidelines (NVL, S3) on major depression, bipolar disorder, ADHD and suicidal behaviour (aided in developing guidelines); board member of DGBS, DGPPN, ECNP and German Depression Foundation, Speakers bureau of: (and participated in advisory boards over the last 3 years) for Cyclerion, Janssen, Medice, SAGE/Biogen and Shire/Takeda; received speaker’s honoraria from Das Fortbildungskolleg; , A. E. Anıl Yağcıoğlu Grant / Research support from: Participated as an investigator for Janssen, Speakers bureau of: (and participated in advisory boards over the last 3 years) for Janssen and Abdi İbrahim Otsuka, A. Luts Speakers bureau of: (or participated in advisory boards for or participated as an investigator) for Janssen-Cilag, Asarina Pharma, Bristol Meyer Squibb, Dr August Wolff GmbH & Co, Eli Lilly, Lundbeck, Pfizer, Allergan, Sunovion and Regeneron., T. Messer Consultant of: National Care Guidelines (NVL, S3) on major depression (aided in developing guidelines), Speakers bureau of: (and participated in advisory boards) for Janssen-Cilag and Otsuka/Lundbeck, R. Nielsen Consultant of: Board member of DSAL and IGSLi, Speakers bureau of: (or participated in advisory boards, received research funds or participated as investigator over the last 3 years) for Boehringer Ingelheim, Compass Pharmaceuticals, Janssen-Cilag, Lundbeck, Otsuka, Sage and Teva Pharmaceuticals, J. Buyze Employee of: Janssen, T. Ito Employee of: Janssen, Y. Kambarov Employee of: Janssen, S. Mulhern Haughey Employee of: Janssen, B. Rive Employee of: Janssen, I. Usankova Employee of: Janssen, C. von Holt Employee of: Janssen, Y. Godinov Employee of: Janssen

